# In vivo assessment of the Optipen optical reflectometer for detecting enamel surface changes following an erosive challenge

**DOI:** 10.1007/s00784-026-07100-6

**Published:** 2026-08-25

**Authors:** Ana Luiza Bogaz Debortolli, Franciny Querobim Ionta, Ana Laura Marques Xavier, Carolina Ruis Ferrari, Thiago Saads Carvalho, Adrian Lussi, Marília Afonso Rabello Buzalaf, Daniela Rios

**Affiliations:** 1 Department of Pediatric Dentistry, Orthodontics and Public Health, Bauru School of Dentistry, University of São Paulo, Bauru, SP Brazil; 2 Department of Biological Sciences, Bauru School of Dentistry, University of São Paulo, Bauru, SP Brazil; 3 Department of Restorative, Preventive and Pediatric Dentistry, School of Dental Medicine, University of Bern, Bern, Switzerland; 4 Department of Operative Dentistry and Periodontology, University Medical Centre Freiburg, Freiburg, Germany

**Keywords:** Dental Erosion, Tooth Enamel, Optical Devices, Dental Diagnostic Techniques, Randomized Controlled Trial

## Abstract

**Objectives:**

To evaluate whether the Optipen optical reflectometer can detect enamel surface changes associated with erosion under clinical conditions.

**Materials and methods:**

In this short-term parallel randomized clinical trial, 24 healthy participants were allocated to two groups: Test (T-rinse with a cola beverage for 30 s) and Control (C-rinse with water). Surface reflectance was measured using the Optipen optical reflectometer on the maxillary central incisors at baseline, immediately after rinsing, and after 2 h of salivary exposure, and expressed as the percentage of surface reflection intensity.

**Results:**

The test group showed significantly lower reflectance values immediately after the erosive challenge (65.4) compared with the control group (102) (*p* < 0.05). After 2 h of salivary exposure, no significant differences were observed between groups (T = 85.7 and C = 97.8).

**Conclusions:**

The Optipen optical reflectometer detected immediate changes in enamel surface reflectance in vivo following a single erosive challenge.

**Clinical relevance:**

This device may be useful for detecting immediate enamel surface changes following erosion. Further studies are needed to optimize reflectance measurements after salivary exposure.

## Introduction

Tooth wear involves the interaction of different mechanisms, including attrition, abrasion and erosion [1]. Erosive tooth wear is defined as the loss of mineralized tooth substance in which dental erosion represents the primary etiological factor [1]. Dental erosion is characterized by the chemical loss of dental hard tissue caused by exposure to acids not derived from oral bacteria. This process initially leads to enamel softening and may progress to irreversible surface loss if the erosive challenge persists [1]. Early detection of enamel alterations is therefore essential for understanding disease progression and evaluating preventive strategies.

Several methods have been proposed to monitor erosive tooth wear. However, clinical studies evaluating preventive strategies remain challenging because measurable tissue loss often requires long observation periods [2]. Techniques such as intraoral scanning allow the longitudinal assessment of tooth surface loss; however, their sensitivity for detecting very early erosive changes remains limited because they rely on measurable tissue loss, whereas the earliest stages of erosion are characterized by subtle surface alterations before tissue loss becomes detectable [3]. Therefore, the development of methods capable of detecting these early enamel surface alterations is essential.

Optical Reflectometer has emerged as a promising approach developed for detecting surface changes associated with dental erosion [4, 5]. Previous in vitro studies demonstrated that reflectometry can identify early enamel alterations and correlate with established measures of erosion [4]. More recently, the reflectometer has been applied in vivo to evaluate preventive treatments; however, this study was limited by experimental conditions that did not fully reproduce the oral environment, as the acid challenge was applied locally to the tooth using a pipette and immediately removed for ion release quantification, without salivary influence [6].

Although the Optipen reflectometer has emerged as a promising tool for the early detection of enamel surface changes, further investigation under clinically relevant conditions is required before it can be applied in short-term randomized clinical trials to screen preventive therapies for dental erosion. Therefore, the aim of this study was to evaluate whether the portable optical reflectometer Optipen can detect enamel surface changes associated with an erosive challenge in vivo under clinical conditions.

## Methods

The study protocol was approved by the Research Ethics Committee (CAAE 75102423.9.0000.5417) and registered in the Brazilian Registry of Clinical Trials (ReBEC: RBR-283VNM6). All volunteers participated after reading and signing the Informed Consent Form.

This study was designed as a parallel, randomized, blinded clinical trial following the CONSORT guidelines to evaluate the ability of the portable reflectometer to detect initial enamel erosion. Participants were randomly allocated in a 1:1 ratio to two groups: test group (erosive challenge with Coca-Cola^®^) and control group (rinse with water). The randomization sequence was generated using Microsoft Excel, and allocation concealment was ensured using sealed envelopes prepared before the beginning of the study. The sample consisted of 24 individuals (12 per group). Sample size calculation was based on data from Pela et al. (2023), considering an estimated standard deviation of 13.4 and an expected between-group difference of approximately 18% under similar experimental conditions, with α = 0.05 and β = 0.20.

Healthy volunteers aged 18–30 years of both sexes were included. Exclusion criteria were smoking, use of medications affecting salivary flow, pregnancy, and systemic diseases. Regarding oral health conditions, only participants without enamel alterations, erosive tooth wear, active caries, gingivitis, periodontitis, or restorations/fixed appliances on the upper anterior teeth were included. Individuals with risk factors for erosive tooth wear (ETW), such as high acid consumption, swimming habits, or gastric disorders, were also excluded. Salivary flow inclusion criteria (≥ 0.3 mL/min unstimulated; ≥1.0 mL/min paraffin-stimulated) were assessed at 8:00–10:00 a.m. to minimize circadian variation [7].

**Fig. 1:**
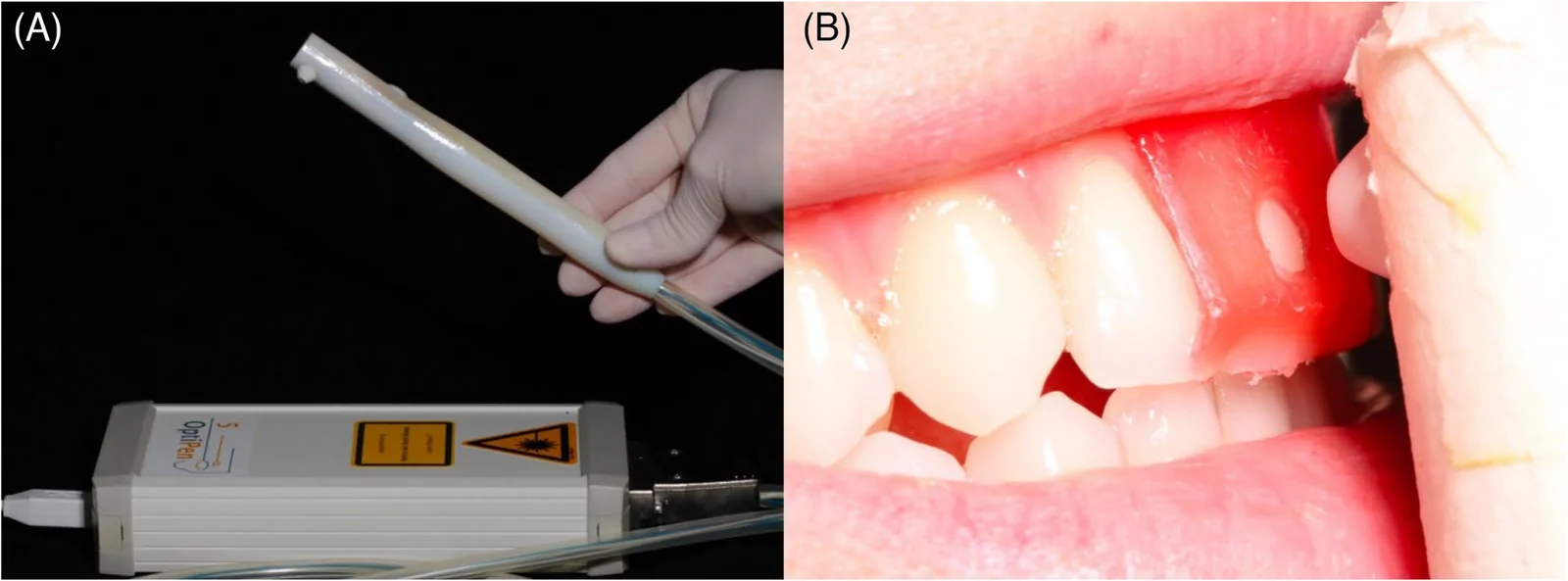
**(A)** Optipen optical reflectometer used for enamel surface reflectance measurements; **(B)** Intraoral application of the device on the vestibular surface of a maxillary central incisor, using a customized wax positioning device to standardize measurement location and angulation

Upon arrival at the clinic, the volunteers underwent professional prophylaxis using pumice and a rubber cup. A customized wax positioning device was then used to standardize the positioning of the Optipen reflectometer on the vestibular surface of both maxillary central incisors throughout the study. The examiner responsible for the measurements was previously calibrated. At each evaluation, the reflectometer was positioned in the standardized location with its tip in gentle contact with the enamel (Fig. 1). Small angular adjustments were then performed within this predefined position until the highest reflectance value was obtained. The highest reflectance value was recorded for each tooth, and the mean value of both maxillary central incisors was used for the statistical analyses.

All measurements were performed in the morning. The envelopes were opened immediately before each procedure by the researcher responsible for administering the treatments. All reflectance measurements were performed by a second researcher blinded to treatment allocation. The individuals in the test group were subjected to the erosive challenge, consisting of rinsing with 30 mL of Coca-Cola^®^ (pH 2.4) for 30 s, directed to the anterior region of the mouth. Participants in the control group rinsed with deionized water (pH 6.8) under the same conditions. Immediately after rinsing, a second surface reflection intensity measurement was performed, followed by a third evaluation two hours after the challenge. During the 2-hour interval, participants refrained from consuming any food or beverages.

Reflectance values were expressed as percentage of surface reflection intensity using the formula %SRI = (IRSf / IRSi) × 100, where IRSi represents the baseline reflection intensity and IRSf the reflection intensity measured at each time point. Data were tested for normality and homogeneity of variance. As these assumptions were satisfied, repeated measures ANOVA followed by Tukey’s post hoc test was applied (*p* < 0.05). Statistical analyses were performed using STATISTICA software (version 11.0; StatSoft Inc.).

## Results

Twenty-four participants (50% female and 50% male, with a mean age of 22.75 for the control group and 23.75 for the experimental group) were randomized and completed the study. No participants were lost to follow-up or excluded after randomization, and all randomized participants were included in the final analyses.

Table 1 presents the percentage of surface reflection intensity (%SRI) for the control and test groups immediately after the rinse and after 2 h. Immediately after the erosive challenge, %SRI values were significantly lower in the test group than in the control group (*p* = 0.006). After 2 h, no statistically significant difference was observed between the groups.

**Table 1:** Baseline surface reflection intensity (SRI) and percentage changes in surface reflection intensity (%SRI) in the control and test groups immediately after the rinse and after 2 h

Intervention	SRI initial	%SRI immediately	%SRI 2 h
Control	4,56 ± 0,57	102 ± 15.5^a, A^	97.8 ± 20.0^a, A^
Test	5,63 ± 1,4 †	65.4 ± 13.9^b, B^	85.7 ± 17.2^a, A^

*Values are presented as mean ± standard deviation (SD). Different lowercase superscript letters indicate statistically significant differences between groups at the same time point (columns), while different uppercase superscript letters indicate statistically significant differences over time within the same group (rows) (repeated-measures ANOVA followed by Tukey’s test, *p* < 0.05)

^†^Significant difference in baseline surface reflection intensity (SRI initial) between groups (independent samples t-test, *p* < 0.05)

Within the control group, %SRI values remained stable over time. In contrast, the l test group showed an increase in %SRI from the immediate post-challenge measurement to the 2-hour evaluation.

## Discussion

The present findings support the responsiveness of the Optipen reflectometer for detecting early surface optical changes under clinical conditions. A significant reduction in reflectance was detected immediately after exposure to the cola beverage. This finding indicates that the reflectometer is sensitive to early surface alterations caused by acid exposure. During the initial stages of dental erosion, the outer enamel layer undergoes superficial softening and increased surface roughness as minerals are dissolved from the surface. These structural changes modify the optical properties of enamel and reduce specular reflection intensity. Previous validation studies have also demonstrated associations between reflectance changes and erosion-related parameters, including surface hardness reduction, calcium release, and surface roughness [4]. However, the present clinical study was not designed to revalidate these associations in vivo, as no validated non-invasive reference method is currently available to directly quantify immediate mineral loss after an erosive challenge in humans [8]. Therefore, the reduction in reflectance observed in the present study should be interpreted as an optical surface response associated with an erosive challenge rather than as a direct measurement of demineralization or evidence of diagnostic validation of the device.

Although erosive tooth wear is a chronic and multifactorial condition involving repeated chemical and mechanical challenges over time, the present model was not designed to reproduce disease progression. Instead, this controlled short-term challenge was selected as an initial step to determine whether the Optipen could detect early surface responses under clinical conditions. This approach may support future short-term clinical screening of preventive or therapeutic strategies before conducting longer and more complex clinical trials.

The stability of reflectance values observed in the control group further reinforces the reproducibility of the measurements obtained with the device. When no erosive challenge was applied, the optical signal remained consistent throughout the experimental period, suggesting that the reflectometer provides reliable readings when the enamel surface remains unchanged. Similar findings have been reported during the validation of optical reflectometry for assessing enamel erosion, where reflectance changes were shown to correlate with established markers of mineral loss and surface modification [4]. However, the present findings should be interpreted as evidence of measurement responsiveness rather than formal reliability or reproducibility, as repeatability parameters were not evaluated in this study.

In contrast, the group that rinsed with a cola beverage showed an increase in reflectance values after two hours. Although this observation could initially be interpreted as a result from mineral deposition from saliva onto the eroded enamel surface [9], there is limited evidence that remineralization processes occurring within two hours of salivary exposure would be sufficient to produce measurable changes in enamel optical properties. A more plausible explanation is the formation of the acquired pellicle, a protein-rich salivary layer known to rapidly adsorb onto dental surfaces [10]. This pellicle may partially fill surface irregularities caused by erosion, leading to a smoother appearance and increased reflectance values [11]. Pellicle-covered enamel may exhibit reduced tissue loss after acidic challenges despite remaining softened, indicating that the presence of the AEP does not necessarily imply complete structural recovery of the underlying enamel [12]. Therefore, the reflectance signal may be influenced not only by enamel alterations but also by biological surface-related factors, particularly acquired enamel pellicle formation. This finding highlights the need for future studies investigating approaches to minimize or control pellicle-related interference in optical measurements.

The study emphasizes the importance of innovative methods for assessing early changes in enamel, contributing to the development of more efficient approaches for studying erosive processes and preventive strategies. The potential relevance of the Optipen is not as a independent diagnostic tool for erosive tooth wear, but as an objective method for detecting early optical surface changes in clinical research settings. Furthermore, the compact design of the device and its relatively simple operation after appropriate training may facilitate its implementation in short-term clinical trials.

Despite these promising findings, some limitations should be acknowledged. Tooth erosion rarely occurs as an isolated process in the oral environment and is frequently associated with mechanical challenges such as abrasion and attrition [1]. In addition, the susceptibility of enamel to erosive demineralization is influenced by intrinsic factors such as mineral composition [9], as well as by extrinsic factors, including salivary conditions and the presence and characteristics of the acquired pellicle [10]. Future studies including longer observation periods and combined chemical-mechanical challenges, are necessary to better understand the performance of the reflectometer under clinically relevant conditions.

Within the limitations of this study, the Optipen optical reflectometer was able to detect short-term changes in enamel surface reflectance following an erosive challenge in vivo. Further studies are required to determine whether these changes correlate with clinically relevant stages of erosive tooth wear as well as to clarify the influence of biological factors, such as acquired enamel pellicle formation, on in vivo measurements and its applicability for monitoring preventive strategies in clinical research.

## Data Availability

All data generated or analyzed during this study are included in this article and its supplementary material files. Further enquiries can be directed to the corresponding author.
